# Radiation Dose-Induced Carotid Artery Stenosis and Brain Necrosis in Head and Neck Cancer—A Real World Cohort Study

**DOI:** 10.3390/cancers16172982

**Published:** 2024-08-27

**Authors:** Henry W. C. Leung, Shyh-Yau Wang, Cheng-Li Lin, Agnes L. F. Chan

**Affiliations:** 1 An-Nan Hospital, China Medical University, Tainan 709, Taiwan; 070506@tool.caaumed.org.tw; 2 College of Medicine, China Medical University, Taichung 404, Taiwan; 0021467@tool.caaumed.org.tw; 3 Kaohsiung Show Chwan Memorial Hospital, Kaohsiung 821, Taiwan

**Keywords:** head and neck cancer, carotid artery stenosis, brain necrosis, nasopharyngeal cancer, radiation therapy

## Abstract

**Simple Summary:**

Radiotherapy is the main local treatment for head and neck cancer. Radiation oncologists and patients should be alert to late carotid artery stenosis and brain necrosis after treatment.

**Abstract:**

**Objective:** This study aims to examine whether radiation therapy doses are related to incidences of carotid artery stenosis and brain necrosis in a large-scale real-world database. **Methods:** We identified a cohort of HNC patients from the catastrophic illness patient dataset using ICD-9 or ICD-10 to compare the incidence and risks of carotid artery stenosis (CAS) and brain necrosis (RIBN) in patients who received a radiation therapy dose of ≥5400 cGy/30 fractions (group A) with those who received a radiation therapy dose of <5400 cGy/30 fractions (group B). The incidence and hazard ratios were quantified using Cox proportional hazards models. **Results:** A total of 19,964 patients were identified in group A and group B. Among them, 965 and 863 cases of CAS and 435 and 359 cases of RIBN were identified in group A and group B, respectively. There was no statistically significant association between the two groups for CAS risk, whereas there was a statistically significant association between the two groups for RIBN risk. The most common primary site of head and neck cancers was the nasopharynx (1144 of 19,964, 5.73%). **Conclusions:** Our study suggests that RT may increase the risk of carotid stenosis and brain necrosis in patients with NPC. To ensure patient safety during treatment, the optimal balance between tumor control and toxicity prevention in individual patients through minimization of the radiation dose to all relevant OARs must be properly understood.

## 1. Introduction

Head and neck cancer (HNC) is one of the most common cancers, with approximately 878,348 new cases and 444,347 deaths worldwide in 2020 [1]. In Taiwan, there were 8277 new cases and 3137 deaths in 2020. The five-year survival rate of head and neck cancer (HNC) after radiation therapy (RT) is about 35% to 89% [2].

Squamous cell carcinoma usually occurs on the lower surface of the oral cavity, pharynx and larynx in the mucosa with an incidence rate of about 90% of cases. Surgical resection, radiotherapy or both have been the usual treatment methods over the past few decades [3,4,5]. More than half of HNC patients require cervical irradiation because of the abundant lymphatic drainage in the neck. Definitive radiotherapy is the primary local treatment for unresectable HNC tumors, either for organ preservation or for the treatment of unresectable HNC tumors [6,7,8]. Surgical resection is often used in the early stages of cancer, but radiotherapy is increasingly considered to replace the need for surgical resection [9,10,11]. For locally advanced cancer, radiotherapy can be used concurrently with chemotherapy or as adjuvant radiotherapy after surgical resection [5].

The prevalence of >50% and >70% carotid stenosis and carotid occlusion after irradiation is 4–25% in patients with HNC who receive radiation therapy, and the cumulative incidence increases over time. In treating HNC, the RT dose is up to 70 Gy/fraction, which can damage the carotid artery, leading to stenosis [12]. Likewise, these patients have at least twice the risk of cerebrovascular events compared with the normal population [12]. Radiation-induced brain necrosis (RIBN) and carotid artery stenosis (CAS) are more serious complications and can potentially lead to cognitive dysfunction, seizure, headache, transient ischemic attack (TIA) or ischemic stroke, and limb paralysis [13,14,15]. In recent years, with the advancement of radiation therapy (RT) technology and comprehensive treatment, patient survival has been extended, but radiation-related late toxicity has also increased and has attracted increasing attention.

Several studies have reported that with the widespread use of intensity-modulated radiation therapy (IMRT), the incidence of RIBN is only 0.13–2.8%, but this is dose-related [13,14]. A recently published study showed that the RIBN incidence rate is 5% over 5 years and 50% over 5 years; the maximum dose (Dmax) is 69.59 Gy and 76.45 Gy, respectively [15]. Therefore, brainstem necrosis is associated with high-dose irritation. The maximum dose is the most important predictive dosimetric factor for RIBN. Dmax < 69.59 Gy is the recommended brainstem limiting dose [15]. In addition, other recently published studies reported that radiation-induced temporal lobe necrosis is one of the late complications after radiation therapy in patients with nasopharyngeal carcinoma. Its incidence also depends on the dose and RT technique [16,17]. However, there are limited studies reporting the risk of brain necrosis and the updated risk of CAS after radiation therapy, so we conducted this study using recent NHIRD big data to examine whether the incidence of CAS and RIBN is associated with radiation therapy dose and other risk factors. In addition, it is important to remind young radiation oncologists of such delayed radiation-related toxicities and develop effective new strategies to prevent them earlier.

## 2. Methods

### 2.1. Data Source and Study Design

This was a retrospective population-based cohort study analyzing data retrieved from the catastrophic illness patient dataset (HV) of the Taiwan National Health Insurance Research Database (NHIRD) from 1 January 2008 to 31 December 2020 [18]. The NHIRD contains comprehensive information on the healthcare utilization of Taiwan National Health Insurance beneficiaries. The analyzed database of all claims was de-identified and all information with patients’ identifiers in the NHIRD was double-encrypted to protect patient confidentiality. The health insurance database was provided for research only. This study was approved by the Research Ethics Committee of China Medical University and Hospital (CMUH109-REC2-031(CR-3)).

### 2.2. Study Population

We identified patients with a diagnosis of head and neck cancers, including lip, oral cavity, larynx, oropharynx, nasopharynx and hypopharynx cancers (based on ICD-9 codes 161 and 140.0–149.9; ICD-10: C76, C00-C14), from January 2008 to December 2020. All patients investigated in this study received IMRT radiotherapy. We divided the cohort of patients into two groups: those received a radiation dose (RT) ≥5400 cGy/30 fractions and those who received a dose <5400 cGy/30 fractions. Based on the literature review and the local radiation oncologist’s clinical experience, the minimum dose to control clinically negative necks (cN0) is 5400 cGy/30 fractions [19,20]. According to NCCN guidelines, the intent of radiotherapy in our country is as a definitive radiation therapy [21]. The cohort was further identified after 1:1 propensity score matching by sex, age, the index date, comorbidities and Charlson comorbidity index (CCI). The index date was defined as the date on which head and neck cancer patients received their first radiation therapy prescription after diagnosis. Patients were excluded if they had a diagnosis of CAS or RIBN or received radiation therapy before the index date. We also excluded patients who were <20 years old and followed-up for less than 1 year.

### 2.3. Main Outcome and Potential Confounding Factors

The primary outcome of this study was the incidence of carotid artery stenosis (ICD-9: 437, 427.8; ICD-10: I65.21–I65.29, I67.2, I77.1) or brain necrosis (ICD-9: 437.8, ICD-10: G46.3–G46.8) after radiation therapy. We also assessed the potential confounding factors that occurred before the index date, including patient demographic and clinical features, such as age and comorbidities, including hyperlipidemia (ICD-9: 272, ICD-10: E78), hypertension (ICD-9: 401–405, ICD-10: I10–I15), cerebrovascular events (ICD-9: 437.9, ICD-10: I67.9), coronary artery disease (ICD-9: 410–414, ICD-10: I10–I16), atrial fibrillation (ICD-9: 427.3, ICD-10: I48.9), congestive heart failure (ICD-9: 428, ICD-10: I50), myocardial infarction (ICD-9: 410, ICD-10: I21,I22), peripheral artery disease (ICD-9: 443.9, ICD-10: I73.9) and smoking status (ICD-9: 305.1, V15.82, ICD-10: F17.2, Z87.891). The Charlson comorbidity index was also used to assess the influence of the comorbidities on the survival rate. We further linked to the Taiwan Cancer Registry database to identify radiation doses and determine the tumor grade of HNC types. The tumor grades defined by the National Cancer Institute are grade X: grade cannot be assessed (undetermined grade); grade 1: well differentiated (low grade); grade 2: moderately differentiated (intermediate grade); grade 3: poorly differentiated (high grade); and grade 4: undifferentiated (high grade) [22]. The higher the grade, the more abnormal the cells look, the faster they are likely to grow and spread, and the poorer the prognosis.

### 2.4. Statistical Analysis

We used chi-square tests to compare patient characteristics between the two groups and *t*-tests to estimate the mean age of patients. The incidence rate was calculated in units of 1000 person-years. Cox proportional hazards regression models were used to estimate the hazard ratios (HRs) for the risk of CAS and RIBN between the two groups. The Kaplan–Meier method was used to estimate the cumulative incidence of CAS and RIBN in the two groups, and the log-rank test was used to compare the differences. All statistical analyses were conducted using SAS 9.4 (SAS Institute Inc., Cary, NC, USA) and R (version 4.0.3 for Windows R). Differences were considered statistically significant if *p* < 0.05.

### 2.5. Sensitivity Analyses

Sensitivity analyzes were performed to test the main outcomes of CAS and RIBN incidence. The first scenario reruns the model in patients who received radiation therapy and those who did not. The procedure for testing the second scenario of RIBN patients is the same as that for the first scenario.

## 3. Results

**Figure 1:**
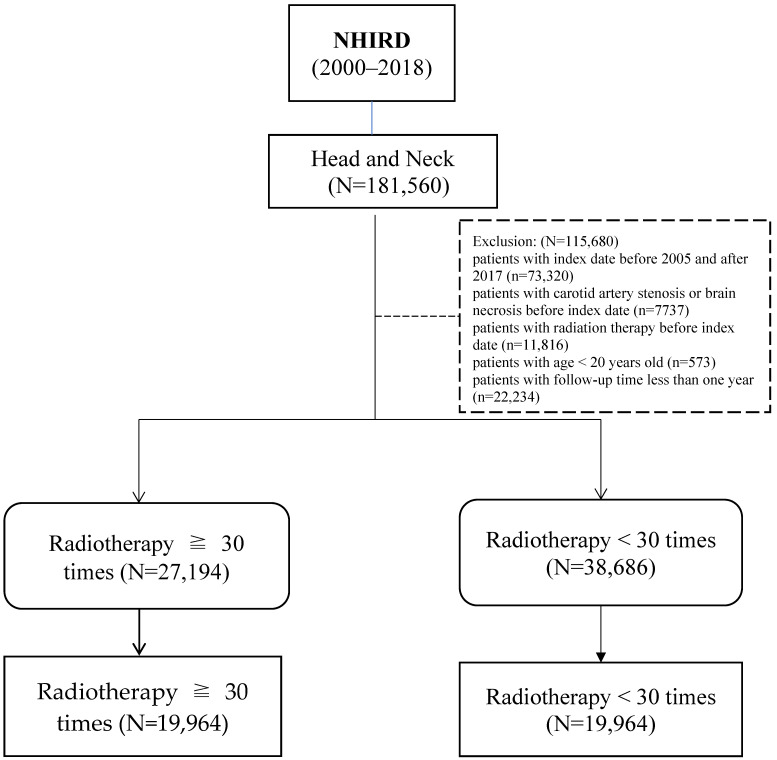
Flow chart of participants selection and study design. RIBN, radiotherapy induced brain necrosis Dose ≥ 30 times (≥5400 cGy/30 fraction).

A total of 27,787 patients with HNC aged ≥20 years were identified as the eligible study cohort (mean age 54.86 years). After propensity score 1:1 matching, the cohort was divided into two groups. A total of 19,964 patients with HNC who received a radiation therapy dose of ≥5400 cGY/30 fractions and 19,964 patients who received a radiation therapy dose of <5400 cGY/30 fractions were identified and defined as group A and group B, respectively (Figure 1). The daily therapeutic dose/fraction in most of the patients was 180 cGy/fraction for 30 days and 200 cGy/fraction for 27 days. The radiation’s biologically effective dose (BED) of patients in groups A and B was 63.72 Gy and 64.80 Gy, respectively [23]. Patient characteristics between the two groups did not show significant differences, except for primary site and cancer grade (Table 1). The incidence rate/1000 person-years was 8.57 in group A versus 8.61 in group B. In a multivariate regression analysis model, a non-significantly increased risk of CAS in group A compared with group B was observed (aHR = 1.05; 95% CI 0.95, 1.16, *p* = 0.329). For RIBN, group A had a significantly increased risk of developing RIBN than group B (aHR = 1.29, 95% CI: 1.11–1.5, *p* < 0.001) (Table 2). 

**Figure 2:**
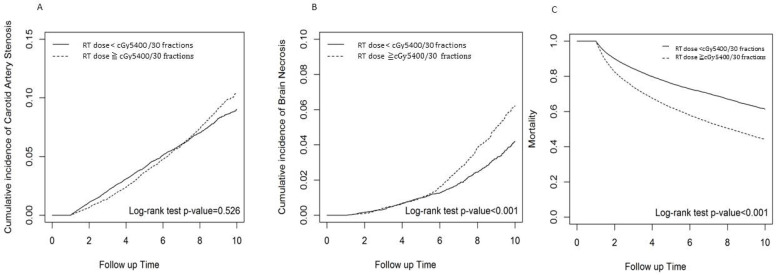
Cumulative incidence of carotid artery stenosis and brain necrosis among head and neck patients with radiation doses cGy5400 ≥ 30 fractions vs. < cGy5400/30 fractions. Incidence rate of (**A**) carotid artery stenosis; (**B**) radiation-induced brain necrosis; (**C**) overall mortality rate.

In the data in Table 3, we found significant differences between the patients that received radiation therapy at doses ≥5400 cGy/30 fractions compared with those that received radiation therapy at doses <5400 cGy/30 fractions. In a multivariate regression analysis model adjusting for all potential clinical confounders, which showed that male patients, age between 20 and 59 years and a follow-up period longer than 6 years were associated with a significantly increased risk of CAS and RIBN. Among the comorbidities, hyperlipidemia and hypertension showed statistically and non-statistically significantly higher risks of RIBN and CAS in patients who received radiation therapy at doses ≥5400 cGy/30 fractions compared with patients who received radiation therapy at doses <5400 cGy/30 fractions. Patients who received radiation therapy at a dose of ≥5400 cGy/30 fractions had a significantly and non-significantly higher cumulative incidence of RIBN or CAS and a higher overall mortality rate than those whose dose was <5400 cGy/30 fractions (Figure 2).

## 4. Sensitivity Analysis

**Figure 3:**
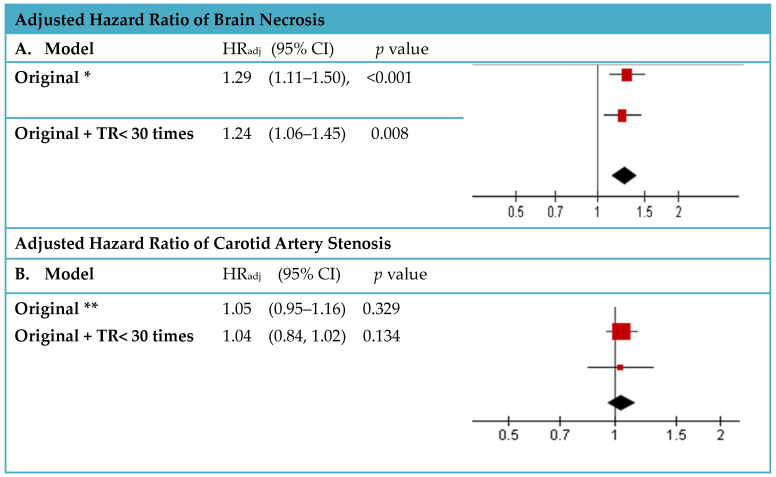
Sensitivity analysis. (**A**) Adjusted hazard ratio of brain necrosis. (**B**) Adjusted hazard ratio of carotid artery stenosis. Remarks: * Adjusted for sex, age, comorbidities, CCI category and primary site of head and neck cancer, tumor grade. ** Adjusted for sex, age, CCI category, comorbidities, primary site of Head and neck cancer, tumor grade. HRadj: adjusted hazard ratio.

Sensitivity analyses in two different scenarios showed similar results for CAS and RIBN compared with the original scenarios (CAS: aHR, 1.04; 95% CI, 0.84, 1.02; RIBN: aHR, 1.24; 95% CI, 1.06 to 1.45) (Figure 3). The results were robust.

## 5. Discussion

To the best of our knowledge, this is the first study to examine the relationship between the radiation therapy dose and radiation therapy-induced late toxicities in a large real-world claim database. The results of our study show significantly increased risk of RIBN between patients that received an RT dose of ≥5400 cGy/30 fractions and of <5400 cGy/30 fractions. This result may be supported by recently published studies showing that the incidence of RIBN depends on the RT technique and dose. They concluded that a radiation dose of 66 to 70 Gy is required for the gross tumor volume (GTV) and 54 to 60 Gy is required for the clinical target volume (CTV). The CTV covers the microscopic, unimaginable extent of tumor spread. Since some types of HNC, such as NPC, often invade the skull base, the temporal lobe (TL) is inevitably included in the radiation field, and some vital structures receive more than 60 Gy of radiation [16]. For CAS, a recently published study may support our choice of ≥5400 cGy/30 fraction as the study grouping criterion. They recommended the use of dose restriction to the carotid artery in IMRT replanning, reducing the mean carotid artery dose to 54 Gy [20].

Another important factor associated with a significantly increased risk of CAS and RIBN is the time interval between radiation therapies. Cheng et al. and Carpenter DJ et al. showed that patients who received treatment for more than 5 years and 6.5 years had greater risk of having carotid artery stenosis (CAS) than those with fewer than 5 years and 6.5 years of follow-up, with an overall prevalence of 21% [12,24,25]. These studies can serve as supporting evidence for our research. We observed a significantly increased risk for patients whose follow-up interval of radiation therapy was more than 6.0 years (72 months). The mean interval of follow-up of over 6 years is also consistent with the recently published literature of Taiwanese Data [12]. Therefore, routine screening is recommended for patients who have received radiation therapy for more than 5 years.

Meanwhile, the RIBN latency reported in our study was longer than that reported in the recently published studies performed by Fan X et al. [15]. They reported that the median time to RIBN after treatment was 28.5 months (2.3 years), but the incidence rate was almost the same (1.25% versus ours, 1.249%). The difference may be due to the earlier use of modern IMRT techniques in Taiwanese hospitals. This low incidence of RIBN may be attributable to the advantages of IMRT, including dose gradient changes and brain hotspots. Since the target area in the treatment plan of nasopharyngeal carcinoma is close to many important organs, how to focus the hotspot on the surface of the brain lesion and whether the dose distribution conforms to the anatomical contour may depend on the quality of radiation therapy planning by experienced radiation oncologists or the development of consensus guidelines to standardize contouring [26]. We believe that the maximum tolerated dose of IMRT to the brain injury lesion and the dose distribution planning technique that conforms to the anatomical contour are also the most important parameters for the induction of RIBN. Comprehensive identification and delineation of organs at risk are critical to the quality of radiation therapy planning and the safe delivery of treatment. Furthermore, our results for clinical confounders were similar to those of previous studies, such as host-related (i.e., age, smoking, hypertension, diabetes) and tumor-related factors (i.e., tumor grade), which were reported to be associated with RIBN [15,27].

RIBN and progressive CAS are serious complications in HNC patients receiving radiation therapy. Due to the lack of effective treatment, RIBN was considered progressive and irreversible in the past. In recent years, bevacizumab, nerve growth factors and gangliosides have been used to treat RIBN; they have achieved similar positive results and are considered cost-effective methods [28]. However, because early prevention is better than salvage, an advanced radiological technique, IMRT, has been used to avoid surrounding normal organs or tissues at risk of receiving high-dose radiation or to reduce the maximum dose to brain tissue to avoid or reduce the occurrence of RIBN in clinical daily practice, and the results are remarkable [20]. For CAS prevention, this is used in addition to carotid duplex sonography after radiation therapy to prevent future ischemic stroke [29]. Recently, some researchers have proposed grouping possible toxicities in HNC patients receiving radiotherapy into toxicity domains and constraining doses to a limited set of OAR in all patients to prevent a few toxicities, such as a mean dose to both parotid glands < 25 Gy to reduce the risk of xerostomia or mean dose to pharyngeal constrictors < 50 Gy to reduce the risk of dysphagia [30,31]. A normal tissue complication probability (NTCP) model was developed to achieve an optimal balance between tumor control and toxicity prevention in individual patients by minimizing radiation doses to all relevant OARs simultaneously to prevent many toxicities [32]. Overall, the role of high-precision radiotherapy technology may help to discriminate the risk of side effects from nearby healthy tissues and organs that should be protected or to deliver large, precise radiotherapy doses to smaller tumor areas, such as brachytherapy, intensity-modulated radiotherapy, proton beam therapy, imaging guided radiotherapy, stereotactic radiotherapy, etc. These advanced technologies clearly delineate the OARs in the process of treatment planning contouring [33].

This study has some limitations. First, there is no radiation therapy dose in the research data base, which may limit the results of our analysis. Second, the currently used ICD CODE cannot clearly distinguish the primary site of HNC, resulting in a high proportion of HNC with unspecified primary sites in this study. Third, confounding factors due to treatment planning techniques not documented by the database may limit the interpretation of CAS progression and RIBN. Fourth, advanced cerebral vascular imaging, such as positron emission tomography, computed tomography angiography, magnetic resonance angiography or conventional angiography, is not routinely scheduled as a confirmatory study at the time of CAS progression or RIBN. This may lead to widespread negligence or a lack of awareness among radiation oncologists and is an issue that needs to be addressed in our medical reimbursement coverage. However, these conditions may be offset by the robust results of the sensitivity analysis.

## 6. Conclusions

Our results may provide further evidence for all radiation oncologists and healthcare professionals to implement effective follow-up strategies and develop practical guidelines for HNC patients with high-risk CAS or RIBN after RT. To ensure patient safety during treatment, developing new strategies to reduce doses to organs at risk may prevent the persistence of long-term side effects. These new recommendations are worthwhile and require larger amounts of clinical evidence to evaluate them [31].

## Figures and Tables

**Table 1:** Characteristics of patients with HNC.

Radiotherapy	* Dose < 30 Fractions		* Dose ≥ 30 Fractions		
	N = 19,964		N = 19,964		
Variables	n	%	n	%	*p*-Value
Gender					0.562
female	2943	14.74	2902	14.54	
male	17,021	85.26	17,062	85.46	
Age, years					0.756
20–59	13,524	67.74	13,495	67.60	
60+	6440	32.26	6469	32.40	
Mean, (SD)	54.86	(11.46)	54.89	(11.43)	0.782
**Primary site specified**					<0.001
Oropharynx	108	0.54	280	1.40	
Nasopharyngeal	1144	5.73	3494	17.50	
Hypopharynx	257	1.29	891	4.46	
Larynx	188	0.94	351	1.76	
Oral cavity	7	0.04	18	0.09	
Sinus/Nasal cavity	29	0.15	69	0.35	
Parotid	191	0.96	196	0.98	
Submandibular	71	0.36	78	0.39	
**Primary site unspecified**	17,969	90.01	14,587	73.07	
**Comorbidities**					
Hyperlipidemia	5733	28.72	5696	28.53	0.682
Cerebrovascular events	176	0.88	200	1.00	0.214
Hypertension	7839	39.27	7793	39.04	0.637
Coronary artery disease	3172	15.89	3132	15.69	0.583
Atrial Fibrillation	211	1.06	236	1.18	0.234
Myocardial infarction	367	1.84	346	1.73	0.427
Peripheral artery disease	265	1.33	294	1.47	0.217
Smoking	868	4.35	865	4.33	0.941
**Charlson comorbidity index, CCI**					0.136
0–2	13,821	69.23	13,958	69.92	
3+	6143	30.77	6006	30.08	
**Tumor Grade**					<0.001
1	3036	15.21	1727	8.65	
2	4398	22.03	5594	28.02	
3	808	4.05	1847	9.25	
4	834	4.18	2453	12.29	
X	10,888	54.54	8343	41.79	

Remarks: * Radiotherapy Dose 30 fractions: treatment course of cGy5400/30 fractions for HNC.

**Table 2:** Hazard ratio of carotid artery stenosis/brain necrosis for HNC patients with radiotherapy dose.

Carotid Artery Stenosis						
Variable	N	PY	IR	aHR	(95% CI)	*p*-Value
**Radiotherapy dose**						
<cGy 5400/30 fractions	965	112,077.72	8.61	1.00	(reference)	-
≥cGy 5400/30 fractions	863	100,648.57	8.57	1.05	(0.95, 1.16)	0.329
female	250	35,636.74	7.02	1.00	(reference)	-
male	1578	177,089.55	8.91	1.44	(1.26, 1.65)	<0.001
20–59	950	147,311.82	6.45	1.00	(reference)	-
60+	878	65,414.46	13.42	1.66	(1.51, 1.83)	<0.001
Mean, (SD)				1.03	(1.03, 1.04)	<0.001
**Primary site**						
Oropharynx	13	1525.20	8.52	1.00	(reference)	-
Nasopharyngeal	181	22,256.50	8.13	1.05	(0.58, 1.90)	0.862
Hypopharynx	37	4449.10	8.32	0.94	(0.50, 1.76)	0.836
Larynx	13	1934.67	6.72	0.67	(0.31, 1.44)	0.299
Oral cavity	8	3047.80	2.62	0.32	(0.13, 0.78)	0.012
Other	1576	179,513.01	8.78	0.85	(0.49, 1.47)	0.561
**Comorbidities**						
Hyperlipidemia	694	58,765.59	11.81	1.17	(1.06, 1.30)	0.002
Cerebrovascular events	35	1887.18	18.55	1.37	(0.98, 1.92)	0.065
Hypertension	1035	78,685.54	13.15	1.71	(1.55, 1.90)	<0.001
Coronary artery disease	456	32,478.67	14.04	1.12	(0.99, 1.26)	0.070
Atrial fibrillation	35	1952.30	17.93	1.26	(0.90, 1.78)	0.177
Myocardial infarction	26	2589.32	10.04	0.80	(0.53, 1.18)	0.260
Peripheral artery disease	35	2717.43	12.88	1.10	(0.79, 1.54)	0.574
**Smoking**	56	7481.59	7.49	0.97	(0.74, 1.27)	0.838
**Charlson comorbidity index**						
0–2	1251	157,356.67	7.95	1.00	(reference)	-
3+	577	55,369.61	10.42	1.09	(0.99, 1.21)	0.088
**Tumor Grade**						
1	144	24,243.78	5.94	1.00	(reference)	-
2	298	47,113.45	6.33	1.08	(0.88, 1.32)	0.450
3	88	12,068.65	7.29	1.20	(0.92, 1.58)	0.179
4	121	15,524.15	7.79	1.36	(0.98, 1.88)	0.063
X	1177	113,776.25	10.34	1.60	(1.34, 1.91)	<0.001
**Brain Necrosis**						
**Variable**	**N**	**PY**	**IR**	**aHR**	**(95% CI)**	***p*-Value**
**Radiotherapy dose**						
<cGy 5400/30 fractions	359	114,571.10	3.13	1.00	(reference)	-
≥cGy 5400/30 fractions	435	102,141.31	4.26	1.29	(1.11, 1.5)	<0.001
**Sex**						
female	124	36,211.08	3.42	1.00	(reference)	-
male	670	180,501.34	3.71	1.28	(1.06, 1.56)	0.012
**Age, years**						
20–59	503	149,255.57	3.37	1.00	(reference)	-
60+	291	67,456.84	4.31	1.16	(0.99, 1.35)	0.067
Mean, (SD)				1.01	(1.01, 1.02)	<0.001
**Primary site specified**						
Oropharynx	7	1545.29	4.53	1.00	(reference)	-
Nasopharyngeal	111	22,413.71	4.95	0.88	(0.39, 2.00)	0.760
Hypopharynx	18	4470.40	4.03	0.83	(0.35, 1.99)	0.679
Larynx	6	1948.32	3.08	0.73	(0.24, 2.17)	0.565
Oral cavity + Sinus/Nasal cavity + Parotid + Submandibular	10	3051.79	3.28	0.77	(0.29, 2.02)	0.591
**Primary site unspecified**	642	183,282.91	3.50	0.56	(0.26, 1.18)	0.124
**Comorbidities**						
Diabetes	280	60,331.79	4.56	1.16	(1.01, 1.37)	0.040
Hyperlipidemia	281	60,341.79	4.66	1.18	(1.01, 1.39)	0.041
Cerebrovascular events	9	1968.25	4.57	0.93	(0.48, 1.79)	0.822
Hypertension	395	81,100.88	4.87	1.60	(1.37, 1.88)	<0.001
Coronary artery disease	156	33,592.77	4.64	0.94	(0.77, 1.14)	0.519
Atrial fibrillation	16	2013.67	7.95	1.89	(1.14, 3.14)	0.014
Myocardial infarction	10	2635.58	3.79	1.01	(0.53, 1.92)	0.984
Peripheral artery disease	14	2793.47	5.01	1.23	(0.72, 2.09)	0.451
**Smoking**	32	7541.60	4.24	1.47	(1.03, 2.11)	0.033
**Charlson comorbidity index, CCI**						
0–2	570	160,208.35	3.56	1.00	(reference)	-
3+	224	56,504.06	3.96	1.17	(0.99, 1.37)	0.064
**Tumor Grade**						
1	66	24,566.41	2.69	1.00	(reference)	-
2	157	47,527.17	3.30	1.20	(0.90, 1.61)	0.215
3	30	12,242.17	2.45	0.81	(0.52, 1.26)	0.343
4	88	15,598.74	5.64	1.76	(1.12, 2.78)	0.015
X	453	116,777.93	3.88	1.08	(0.83, 1.40)	0.563

Remarks: N, patient number; PY, person-year; IR: incidence rate per 1000 person-years; aHR, adjusted hazard ratio; significant. *p* < 0.05.

**Table 3:** Comparison of CAS or RIBN risks in patients with doses ≥30 vs. ≤30 fractions associated with all covariates.

	Carotid Artery Stenosis				
Radiotherapy Dose	<cGY5400/30 Fractions	≥cGY5400/30 Fractions			
Variables	N	N	aHR	(95% CI)	*p*-Value
**Sex**					
female	153	97	0.70	(0.54, 0.9)	0.006
male	812	766	1.10	(1.00, 1.22)	0.054
**Age, years**					
20–59	474	476	1.17	(1.03, 1.33)	0.015
60+	491	387	0.89	(0.78, 1.02)	0.095
**Primary site specified**					
Oropharynx + Oral cavity + Sinus/Nasal cavity + Parotid + Submandibular	5	16	1.40	(0.50, 3.91)	0.516
Nasopharyngeal	39	142	0.70	(0.48, 1.00)	0.050
Hypopharynx	6	31	1.22	(0.51, 2.92)	0.663
Larynx	5	8	0.73	(0.24, 2.24)	0.581
**Primary site unspecified**	910	666	1.03	(0.94, 1.14)	0.502
**Comorbidities**					
Diabetes	358	334	1.08	(0.91, 1.23)	0.366
Hyperlipidemia	359	335	1.04	(0.90, 1.21)	0.608
Cerebrovascular events	19	16	0.82	(0.42, 1.59)	0.552
Hypertension	556	479	0.98	(0.87, 1.11)	0.754
Coronary artery disease	259	197	0.84	(0.70, 1.01)	0.066
Atrial fibrillation	14	21	1.51	(0.77, 2.97)	0.234
Myocardial infarction	18	8	0.53	(0.23, 1.23)	0.140
Peripheral artery disease	23	12	0.52	(0.26, 1.04)	0.066
Smoking	23	33	1.54	(0.90, 2.65)	0.113
**Charlson comorbidity index, CCI**					
0–2	651	600	1.05	(0.94, 1.18)	0.364
3+	314	263	0.99	(0.84, 1.17)	0.925
**Tumor Grade**					
1	75	69	1.52	(1.10, 2.11)	0.012
2	110	188	1.29	(1.02, 1.63)	0.035
3	26	62	0.96	(0.61, 1.52)	0.853
4	28	93	0.71	(0.46, 1.09)	0.117
X	726	451	0.99	(0.88, 1.12)	0.900
**Follow-up time**					
<6 years	707	558	0.89	(0.79, 1.00)	0.051
6+ years	258	305	1.48	(1.24, 1.76)	<0.001
**Brain Necrosis**					
**Variables**	**N**	**N**	**aHR**	**(95% CI)**	***p*-Value**
**Sex**					
female	57	67	1.33	(0.94, 1.90)	0.112
male	302	368	1.5	(1.29, 1.75)	<0.001
**Age, years**					
20–59	210	293	1.69	(1.42, 2.02)	<0.001
60+	149	142	1.15	(0.91, 1.44)	0.244
**Primary site specified**					
Oropharynx + Larynx + Oral cavity + Sinus/Nasal cavity + Parotid + Submandibular	3	20	2.94	(0.87, 9.94)	0.083
Nasopharyngeal	19	92	0.85	(0.52, 1.41)	0.532
Hypopharynx	3	15	1.22	(0.35, 4.24)	0.751
**Primary site unspecified**	334	308	1.33	(1.14, 1.55)	<0.001
**Comorbidities**					
Diabetes	130	148	1.17	(0.90, 1.50)	0.206
Hyperlipidemia	131	150	1.35	(1.06, 1.7)	0.013
Cerebrovascular events	4	5	1.46	(0.39, 5.46)	0.577
Hypertension	190	205	1.29	(1.06, 1.57)	0.012
Coronary artery disease	81	75	1.09	(0.79, 1.49)	0.608
Atrial fibrillation	13	3	0.22	(0.06, 0.78)	0.019
Myocardial infarction	5	5	1.29	(0.36, 4.62)	0.700
Peripheral artery disease	6	8	1.83	(0.63, 5.35)	0.270
Smoking	15	17	1.41	(0.70, 2.84)	0.335
**Charlson comorbidity index, CCI**					
0–2	248	322	1.57	(1.33, 1.85)	<0.001
3+	111	113	1.25	(0.96, 1.63)	0.092
**Tumor Grade**					
1	31	35	1.72	(1.06, 2.80)	0.028
2	54	103	1.46	(1.05, 2.02)	0.026
3	9	21	0.87	(0.40, 1.89)	0.719
4	17	71	0.77	(0.45, 1.31)	0.335
X	248	205	1.33	(1.10, 1.60)	0.003
**Follow-up time**					
<6 years	165	174	0.89	(0.71, 1.12)	0.333
6+ years	194	261	1.67	(1.38, 2.03)	<0.001

Notes: N: number of patients; HR: hazard ratio.

## Data Availability

The data presented in this study are available on request from the corresponding author due to privacy.

## Abbreviations

RT	Radiation therapy
HNC	Head and neck cancer
CAS	Carotid artery stenosis
RIBN	Radiation-induced brain necrosis
HR	Hazard ratio
CI	Confidence interval
